# SARS-CoV-2 Breakthrough Infections in Health Care Workers: An Italian Retrospective Cohort Study on Characteristics, Clinical Course and Outcomes

**DOI:** 10.3390/jcm12020628

**Published:** 2023-01-12

**Authors:** Luigi De Maria, Stefania Sponselli, Antonio Caputi, Pasquale Stefanizzi, Antonella Pipoli, Gianmarco Giannelli, Giuseppe Delvecchio, Silvio Tafuri, Francesco Inchingolo, Giovanni Migliore, Francesco Paolo Bianchi, Paolo Boffetta, Luigi Vimercati

**Affiliations:** 1 Interdisciplinary Department of Medicine, University of Bari, 70124 Bari, Italy; 2 General Direction, Policlinico Regional Hospital of Bari, 70124 Bari, Italy; 3 Stony Brook Cancer Center, Stony Brook University, Stony Brook, NY 11794, USA; 4 Department of Medical and Surgical Sciences, University of Bologna, 40138 Bologna, Italy

**Keywords:** COVID-19, SARS-CoV-2 vaccination, breakthrough infections, health workers

## Abstract

Background: The aim of this study was to determine the characteristics, clinical course and outcomes of COVID-19 breakthrough infections (BIs) among healthcare workers (HCWs) of an Italian University Hospital. Methods: A retrospective observational study was conducted on 6111 HCWs, from January 2021 to February 2022. The study population was offered the full vaccination with BNT162b2 mRNA COVID-19 vaccine. To allow return to work after BI, the protocol required one negative nasopharyngeal RT-PCR swab followed by a medical examination to assess the HCW’s health status. Laboratory tests, instrumental tests and specialist evaluations were carried out if necessary. Results. The cases of BIs observed numbered 582 (9.7%). The frequency of BIs was significantly higher in females than in males (67% vs. 33%; *p* = 0.03), and in nurses than in all other professional categories (*p* = 0.001). A total of 88% of the HCWs affected by BI were still symptomatic after the negative swab. None of the instrumental tests carried out showed any new findings of pathological significance. All cases showed progressive disappearance of symptoms, such that no cases of long COVID and no hospitalization or deaths were recorded. Conclusions. Our results confirm that SARS-CoV-2 infections occur even after a full vaccination course; however, the clinical course is favorable and severe outcomes are reduced.

## 1. Introduction

Since the start of the COVID-19 pandemic, more than 6 million deaths have been recorded worldwide, of which about 180,000 were in Italy [1].

The biological risk of healthcare workers (HCWs) exposed to severe acute respiratory syndrome coronavirus 2 (SARS-CoV-2) has been a major issue [2]. Monitoring of HCWs through screening protocols is essential to rapidly diagnose and isolate cases of infection and, consequently, to prevent outbreaks of hospital infection and reduce hospital absenteeism of health personnel [3,4,5,6]. In health facilities prevention measures and protocols have been crucial in containing the spread of the infection [7,8]. Rapid vaccine development has had a major impact on clinical outcomes, reducing severe symptomatic disease and virus transmission rates [9,10,11]. As of March 2022, about ten billion vaccine doses have been administered worldwide, and at least one dose has been administered to 63% of the total population [12]. In Italy, a report of the “COVID-19 vaccine surveillance system” of the Italian Ministry of Health showed that the incidence of COVID-19 cases decreases from 1.13 per 10,000 person-days in the first 14 days after the first vaccine dose, to 0.34 after the second dose [13]. On the other hand, not all HCWs have welcomed the COVID-19 vaccine [14,15], and the succession of new variants with greater transmissibility and greater propensity to determine breakthrough infections (BIs) in vaccinated subjects are a cause of particular attention [16]. The incidence of BIs, defined as infections occurring in fully vaccinated people, has been increasingly frequently reported, even though it is associated with asymptomatic and mild diseases [17,18]. In a meta-analysis of more than 50 studies in several countries, vaccine effectiveness against transmission of the infection, hospitalization, and death was 89.1%, 97.2%, 97.4%, and 99%, respectively [19]. However, severe symptomatic cases of BIs have also been reported in the literature, although there are few studies on the clinical course and outcomes of BIs in large populations [20,21].

The aim of this study was to determine the characteristics, clinical course, and outcomes of COVID-19 BI cases among HCWs of the University Hospital of Bari, one of the major COVID-19 hub centers in southern Italy.

## 2. Materials and Methods

### 2.1. Study Design, Setting, and Population

A retrospective observational study was performed at the University Hospital of Bari, southern Italy, on a population of 6111 HCWs, during an observation period from January 2021 to February 2022.

The cohort was included in the European Commission-sponsored Orchestra project.

Vaccine BI was defined as PCR-confirmed COVID-19 ≥ 14 days following the receipt of a second dose of BNT162b2 mRNA COVID-19 vaccine [22]. On 27 December 2020, vaccination of all HCWs was started, and the study subjects were offered full vaccination with the BNT162b2 mRNA COVID-19 vaccine.

HCWs were stratified according to the following job categories: doctors, nurses, technicians, administrative staff and other HCWs (such as biologists and psychologists).

All HCWs had been subjected to continuous follow-up since the beginning of pandemic according to the preventive protocol established by the Operative Unit of Occupational Medicine. All asymptomatic HCWs underwent nasopharyngeal RT-PCR swab for SARS-CoV-2 infection with intervals of about fourteen days. Privileged quick access to molecular testing was guaranteed for close contacts with COVID-19 cases and for subjects with signs and symptoms of the disease. To resume work after BI, all HCWs had to undergo a health surveillance medical examination at the Operative Unit of Occupational Medicine. Laboratory tests, instrumental tests, and specialist evaluations (i.e., pneumological evaluation, cardiological evaluation) were carried out if necessary. The resumption of work after BI was allowed only with a negative nasopharyngeal RT-PCR swab and in the absence of symptoms and signs related to the disease. All the operating units of the hospital were also classified as “low risk of infection units” (LRIUs) or “high risk of infection units” (HRIUs) on the basis of the biological risk assessment from exposure to SARS-CoV-2. High-risk procedures, performed in HRIUs, include the direct assistance to COVID-19 patients, the execution of urgent invasive maneuvers with the possibility of generating aerosols and the manipulation of biological samples.

The oropharyngeal and nasopharyngeal specimens were stored in sterile test tubes and analyzed in the hospital’s virology laboratories. All SARS-CoV-2 diagnostic testing procedures and specimen collection methodologies were performed in accordance with CDC guidelines [23].

For all BI cases, we examined the following characteristics: gender, reason for testing, job, previous SARS-CoV-2 infection, comorbidity, flu vaccines. The clinical course was evaluated on the basis of the symptoms, their duration and any new clinical findings of pathological significance. Severe outcomes such as hospitalizations and deaths were also evaluated.

All subjects were informed that data from the research protocol would be treated in an anonymous and collective way using scientific methods and for scientific purposes in accordance with the principles of the Declaration of Helsinki. Ethical approval was not necessary because all medical and instrumental examinations were performed according to Italian law concerning the protection of workers exposed to occupational risks (D.Lgs. 81/2008). Nevertheless, the study was approved by the Ethics Committee of Azienda Ospedaliero-Universitaria Consorziale Policlinico of Bari (Parere Studio N. 7241).

### 2.2. Statistical Analysis

Continuous variables were expressed as mean and standard deviation. Shapiro–Wilk’s test for distributional normality was performed. Because the variables were normally distributed, Student’s t test was performed to compare the means. Categorical variables are expressed as absolute and perceptual frequencies, and frequency comparison was performed using the chi-square test. A *p* value < 0.05 was considered statistically significant. A univariate logistic regression test was used to calculate Odd Ratios (OR) and their 95% confidence intervals (C.I.).

## 3. Results

A total of 5996 of the 6111 HCWs (98.1%) completed the vaccination cycle. The cases of breakthrough infections observed were 582 (9.7%), 384 (66%) female and 198 (34%) male. Nurses were the most affected (38%), followed by doctors (37%). The most frequent symptoms were cough (49%), nasal congestion (47%), rhinorrhea (42%), myalgia and asthenia (42%). The source of infection was non-occupational in 78% of cases and only 4% had a previous SARS-CoV-2 infection. Table 1 shows the characteristics of BI cases.

Comparison of the BI group with the non-BI group (Table 2) showed that the mean age was not significantly different between the two groups (41.4 y.o. vs. 46.4 y.o.; *p* = 0.223). The frequency of BIs was significantly higher in females than in males (67% vs. 33%; *p* = 0.03), with females having a higher risk of contracting BI (OR = 1.3; CI = 1.097–1.575; *p* = 0.003). The frequency of BIs was significantly higher in nurses than in all other professional categories (doctors, technicians, administrative, others; *p* = 0.001). The frequency of previous SARS-CoV-2 infection in the BI group was lower than in the non-BI group (4% vs. 10%), and the difference was at the limit of statistical significance (OR = 0.67; CI = 0.437–1.019; *p* = 0.059). Moreover, the frequency of BIs was higher among operators in LRIUs than operators in HRIUs. Specifically, 481/4797 (10%) LRIU operators and 101/1199 (8.4%) HRIU operators tested positive. The 83% of overall BIs was observed in LRIUs.

**Figure 1:**
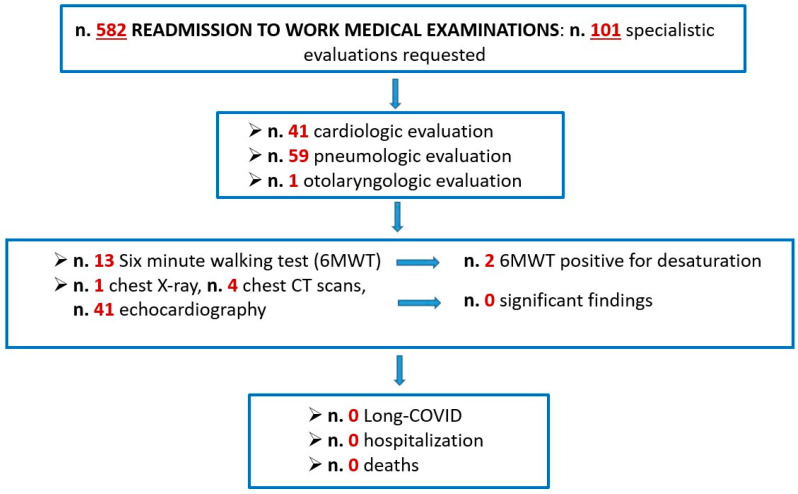
BI readmission to work and outcomes.

All 582 HCWs affected by BI underwent a health surveillance medical examination to allow their readmission to work at the Operative Unit of Occupational Medicine (Figure 1). As a result, 101 specialist evaluations (59 pneumological evaluations, 41 cardiological evaluations and 1 otolaryngological evaluation), 46 instrumental examinations (41 echocardiograms, 4 chest CT scans, 1 chest X-ray) and 13 six-minute walking tests (6MWTs) were requested to identify the persistence of signs and symptoms related to the disease. In particular, 88% of HCWs affected by BI were still symptomatic after the negative swab, at the time of the medical examination for readmission to work. The most frequent signs and symptoms were cough (57%), nasal congestion/rhinorrhea (52%), myalgia/asthenia (49%), headache (33%), palpitations (30%), ageusia/anosmia (16%) and dyspnea (12%). None of the instrumental tests carried out showed any new findings of pathological significance, and only two 6MWTs were positive for desaturation. All cases of symptomatic BI at the time of readmission to work showed progressive disappearance of symptoms in the following days, such that no cases of long-COVID and no hospitalizations or deaths were recorded.

## 4. Discussion

In this study, we aimed to determine the characteristics, clinical course and outcomes of COVID-19 BI cases among HCWs of the University Hospital of Bari, during an observation period from January 2021 to February 2022. The overall frequency of SARS-CoV-2 BIs was 9.7%. A study conducted on the same population of HCWs showed a lower frequency of SARS-CoV-2 infections (6%) in the pre-vaccination phase [24]. This result could be explained by the progressive reduction of government measure on containment and management of epidemiological emergency at national and local level, observed during the vaccination phase, which allowed a higher viral circulation among the general population, despite the availability of vaccines. In support of this hypothesis, in our study, more BIs showed a non-occupational source (78%) compared to an occupational one (19%). Furthermore, the criticality represented by the phenomenon of the continuous emergence of new variants of SARS-CoV-2 must be considered. These emerging “variants of concern (VOCs)” may have mutations in the spike protein that could make vaccination less effective [25,26,27]. Other literature studies have also shown a lower incidence of BIs. In a recent large European multi-center study on a cohort of HCWs, the cumulative incidence of BIs from February 2021 to November 2021 was 1.2% [28]. In the study by Raju Vaishya et al., 85 of 3235 (2.63%) vaccinated HCWs contracted the SARS-CoV-2 infection after vaccination in a study period from 16 January to 24 April 2021 [29]. In both of these studies, however, the study period was shorter than that in our study, and we observed most of the BIs during the last months of the observation period (December 2021–February 2022).

Our results showed that females were more affected by SARS-CoV-2 infection than males (*p* = 0.003), which is in accordance with the gender differences highlighted in other studies [27,29,30]. This result is also in accordance with national-level findings since the pre-vaccination phase [24,31]. Moreover, as for the pre-vaccination phase [20], the frequency of BIs was significantly higher in nurses than in all other professional categories (*p* = 0.001) and was higher among operators in LRIUs than operators in HRIUs, although this result was not statistically significant (*p* = 0.093). This finding, in agreement with other studies, confirms the greater attention by HCWs to the correct rules of PPE use in the departments dedicated to the care of COVID-19 patients [32,33,34].

We observed that most of the cases of BIs (88%) were still symptomatic after the negative swab, at the time of the medical examination for re-admission to work, and the most frequent signs and symptoms were cough, nasal congestion/rhinorrhea, myalgia/asthenia, palpitations. In agreement with our results, Petros Ioannou et al. found a high frequency of mild symptoms in vaccinated subjects, including nasal congestion and rhinorrhea with no fever, indicating that diagnosing COVID-19 in vaccinated healthcare workers may become increasingly more complex [35].

However, despite the large number of specialist evaluations and instrumental test carried out, no new findings of pathological significance were highlighted. All cases of symptomatic BI at the time of readmission to work showed progressive disappearance of symptoms in the following days such that no cases of long-COVID and no hospitalizations or deaths were recorded. This finding is in agreement with other studies reported in the literature. Dagan et al. suggested that the COVID-19 vaccination was highly effective at preventing severe outcomes after infection, causing decreases in hospitalization, severe illness and death [36]. Most of the symptomatic BIs reported by Raju Vaishya et al. did not require intensive care unit admission, and there were no deaths during the study period [37]. Moreover, a study conducted on the same population of HCWs in the pre-vaccination phase reported 199 cases of long COVID in a one-year period of observation [38]. Compared to the 0 cases observed in this study, this finding highlights the role of the vaccine in reducing the duration of symptoms associated with SARS-CoV-2 infection. However, in contrast with our results, Moriah Bergwerk et al. found that 19% of BI cases had long-COVID symptoms (>6 weeks) [39].

Our study had certain limitations. It was a single-center study, and a single vaccine type was used. Furthermore, the genome sequencing and antibody titers were not tested for the entire study population for operational and logistical reasons. Finally, HCWs vaccinated with BNT162b2 were not stratified based on the duration of vaccination. Overall, this study on a large population of HCWs reports the clinical outcomes of the short-term BIs in HCWs.

## 5. Conclusions

This study confirmed that SARS-CoV-2 infections can occur even after a full vaccination course; however, the clinical course is favorable, and severe outcomes are reduced. There was a moderate frequency of COVID-19 (9.7%) in vaccinated HCWs, most of which were symptomatic (88%). Despite the large number of specialist evaluations and instrumental tests carried out, no new findings of pathological significance were highlighted. All cases of symptomatic BI at the time of readmission to work showed progressive disappearance of symptoms in the following days, so no cases of long-COVID and no hospitalizations or deaths were recorded. Female HCWs and nurses were significantly more affected by BIs. The BNT162b2 vaccination seemed to provide protection from severe disease, but our findings raise concerns about lower protection provided regarding transmission. This weakness highlights the need for continuing protective measures to prevent the spread of SARS-CoV-2 infection in hospital settings.

## Figures and Tables

**Table 1:** Characteristics of BI cases.

	N	%
Gender		
M	198	34%
F	384	66%
Symptoms		
Fever	178	31%
Dyspnea	69	12%
Rhinorrhea	243	42%
Cough	283	49%
Myalgia and Asthenia	242	42%
Ageusia	67	12%
Anosmia	81	14%
Pharyngodynia	210	36%
Nasal congestion	271	47%
Gastrointestinal Symptoms	67	12%
Headache	161	28%
Other	58	10%
Reason for testing		
Screening	313	54%
Symptoms	258	44%
Close Contact	11	2%
Job		
Doctors	217	37%
Nurses	220	38%
Other HCWs	101	17%
Technicians	27	5%
Administrative staff	17	3%
Previous SARS-CoV-2 infection	26	4%
Source of infection		
Occupational	113	19%
Non-occupational	456	78%
Unknown	13	2%
Comorbidity	297	51%
Flu vaccines 20-21	346	59%
Flu vaccines 21-22	417	72%

**Table 2:** Comparison between BI group and non-BI group.

	BI						
	YES (n. 582)		NO (n. 5414)				
Vaccine doses Administered *	3 × 576 2 × 6		3 × 5058 2 × 356				
	Mean	S.D.	Mean	S.D.	*T*-test		
Age	41.4	12.4	46.4	12.8	*p* = 0.223		
	n.	%	n.	%	Chi-square test	OR	CI
Gender							
Female	388	67%	3267	60%	*p* = 0.003	1.3	1.097–1.575
Male	194	33%	2147	40%			
Previous Infection	24	4%	558	10%	*p* = 0.059	0.67	0.437–1.019
Job							
Doctors	216	37%	2667	49%	*p* < 0.001		
Nurses	222	38%	1463	27%			
Other HCWs	110	19%	759	14%			
Technicians	21	4%	179	3%			
Administrative	13	2%	346	6%			
Occupational Risk							
HRIU **	101	17%	1098	20%	*p* = 0.093	0.825	0.659–1.033
LRIU ***	481	83%	4316	80%			

* (n. of doses × n. of subjects). ** HRIU: high risk of infection units. *** LRIU: low risk of infection units.

## Data Availability

Data sharing will take place according to the guidelines established within the Orchestra project (www.orchestra-cohort.eu) (accessed on 30 December 2022).
